# Complete Genome Sequence of Burkholderia cenocepacia K56-2, an Opportunistic Pathogen

**DOI:** 10.1128/MRA.01015-20

**Published:** 2020-10-22

**Authors:** Inmaculada García-Romero, Miguel A. Valvano

**Affiliations:** a Wellcome-Wolfson Institute for Experimental Medicine, Queen's University Belfast, Belfast, United Kingdom; University of Southern California

## Abstract

Burkholderia cenocepacia K56-2, an opportunistic bacterium for people with cystic fibrosis (CF), belongs to the Burkholderia cepacia complex (Bcc) and is consistently used as a model pathogen. We describe here the closed genome sequence for this strain, which will help advance research in B. cenocepacia biology and omics studies.

## ANNOUNCEMENT

The Burkholderia cepacia complex (Bcc) comprises a set of *Burkholderia* species that cause respiratory infection in people with cystic fibrosis (CF) (1 – 3). These bacteria are multidrug-opportunistic intracellular pathogens (reviewed in reference 1). B. cenocepacia, in particular, can be transmissible (4) and causes a fatal condition known as cepacia syndrome (5, 6). In this whole-genome sequencing (WGS) project, we have closed the genome sequence of B. cenocepacia K56-2, which belongs to the ET-12 lineage and was isolated from a CF patient in Canada (2, 3). The strain used in this study was isolated as described in reference 3.

To date, no complete genome sequence for K56-2 has been deposited into GenBank; a draft genome containing 19 contigs was published in 2013 (7). In this work, the genome sequence was assembled *de novo*.

Strain K56-2 was grown in 25 ml LB (Melford) at 37°C and 180 rpm to an optical density (600 nm) of 0.6. Pelleted bacteria (4,000 × *g* for 10 min) were resuspended in a cryopreservative (Microbank; Pro-Lab Diagnostics UK, United Kingdom) and shipped to MicrobesNG (Birmingham, United Kingdom). Two different sequencing approaches were used, Illumina HiSeq technology (a 250-bp paired-end protocol) and a GridION system using a FLO-MIN-106 (R.9.4) flow cell (ONT, United Kingdom). For the former, genomic DNA was purified using a QIAamp DNA minikit (Qiagen, Germany). For the latter, high-molecular-weight genomic DNA was extracted using a Nanobind CCB Big DNA kit (Circulomics, MD, USA). Libraries for Illumina sequencing were prepared using the Nextera XT library prep kit (Illumina, San Diego, USA) according to the manufacturer’s protocol. Long-read genomic DNA libraries for GridION sequencing were prepared with the Oxford Nanopore SQK-RBK004 kit (ONT) using 500 ng of high-molecular-weight DNA and in accordance with MicrobesNG protocols (https://microbesng.com/documents/5/MicrobesNG_Methods_Document_-_PDF.pdf). For Nanopore reads, base calling was performed using Guppy v3.0.6+9999d81 (8), generating 29,635 long reads with an *N*_50_ value of 7,943 bp. Illumina reads were adapter trimmed using Trimmomatic 0.30 with a sliding window quality cutoff of Q15 (9), resulting in 1,734,906 reads of 250 bp. Illumina and Nanopore reads were coassembled using Unicycler v0.4.0 (10), obtaining a final assembly of 4 replicons (with a genome coverage of 107.62×). Default parameters were used for all software unless otherwise specified.

The complete genome sequence of strain K56-2 consists of 3 circular chromosomes of 3,673,077 bp (GC content of 66.89%), 3,211,025 bp (GC content of 67.29%), and 765,157 bp (GC content of 66.92%) and a plasmid of 92,661 bp (GC content of 62.76%). The smaller replicon was defined as a plasmid due to the absence of rRNAs and tRNAs and the high similarity (99.9% identity) with pBCJ2315 (NC_011003). All the replicons were manually rotated to match those in the B. cenocepacia J2315 genome (11). Functional annotation was performed using Prokka 1.12 (12), with a cutoff E value of 1e−05 for coding DNA sequence (CDS) annotation plus adding the noncoding RNA (ncRNA) annotation step using Infernal software v1.1.3 (13) and the Rfam database (14).

According to the automatic annotation, the K56-2 genome comprises 7,121 genes, with 6,973 protein-encoding genes, 82 tRNAs, 18 rRNAs, and 48 noncoding RNAs (Table 1).

**TABLE 1:** Main features of K56-2 genome annotation

Replicon	Locus tag	AN^*a*^	No. of:				
			Genes	Proteins	rRNAs	tRNAs	ncRNAs
Chr 1	K562_1	CP053300	3,453	3,337	12	67^*b*^	37
Chr 2	K562_2	CP053301	2,902	2,880	3	11	8
Chr 3	K562_3	CP053302	660	651	3	4	2
Plasmid	K562_4	CP053303	106	105			1
Total			7,121	6,973	18	82	48

a GenBank accession number.

b Including a transfer-messenger RNA (tmRNA).

### Data availability.

The genome sequence of strain K56-2 was deposited in GenBank under the accession numbers indicated in Table 1. The raw sequence reads were deposited in the SRA under the accession numbers SRR11609092 (GridION) and SRR11609093 (Illumina HiSeq 2500). The associated BioProject and BioSample accession numbers are PRJNA627986 and SAMN14693155, respectively.
